# Association of antenatal or neonatal SARS-COV-2 exposure with developmental and respiratory outcomes, and healthcare usage in early childhood: a national prospective cohort study

**DOI:** 10.1016/j.eclinm.2024.102628

**Published:** 2024-05-03

**Authors:** Rebecca Jackson, Rosie Cornish, Zoe Daskalopoulou, Chris Gale, Madeleine Hurd, Samantha Johnson, Marian Knight, Jennifer J. Kurinczuk, Kathryn Woodward, Ela Chakkarapani, SINEPOST collaborative group, Helen Mactier, Elizabeth Draper, Don Sharkey, Cora Doherty, Karen Shorthose, Nagendra Venkata, Claire Cooper, Claire Lee, Louise Coke, Clare Cane, Cynthia Diaba, Sankara Narayanan, Ghada Ramadan, Alys Capell, Dan Jolley, Jennifer Pullen, Rachel Wane, Liz Ingram, Rosaline Garr, Amy Millington, Manal El-Bokle, Paula Brock, Bev Hammond, Matthew Milner, Shalini Ojha, Sarah Miller, Stephanie Grigsby, Susara Blunden, Ruth Shephard, Emma Williams, Balamurugan Thyagarajan, Phillippa Crowley, Kirsty Le Doare, Emily Marler, Ajay Sinha, Nicolene Plaatjas, Dominic Smith, Jennifer Baker, Muhammad Ali, Jennifer Smith, Ranganath Ranganne, Kate Stanbury, Tim Scorrer, Alison LePoidevin, Sharon Westcar, Myrna Maquinana, Clare O'Brien, Seren Willson, Jessica Simkin, Amy Carmichael, Laura Salter, Bhavna Sharma, Caroline Dixon, Janet Brown, Amaryl Jones, Kate Townsend, Emma Tanton, Paul Fleming, Fiona Stacey, Richard Hutchinson, Laura Plummer, Louise Swaminathan, Charu Bhatia, Rebecca Dubber, Jenny Dixon, Angela Phillipson, Julie Groombridge, Tracey Benn, Kathryn Johnson, Lindsay Uryn, Sanjay Salgia, Lisa Frankland, Caroline Salmon, Asharee Green, Elizabeth Lek, Nerea Rodal-Prieto, Julie Grindey, Grainne O'Connor, Afaf Tebbal, Sophie Cullinan, Paula Sugden, Umberto Piaggio, Sarah Farmer, Daisy Tudor, Ambalika Das, Donna Nicholls, Charlotte Lea, Ruth Bowen, Rebecca Mann, Georgina Turner, Chinthika Piyasena, Joanna Robinson, Jain Neeraj, Gillian Godwin, Bridget Oduro, Ramon Fernandez, Kalyana Gurusamy, Liz Pilling, Richard Mupanemunda, Sarah Didier, Jessica Ellis, Anitha James, Sandie Bohin, Linda Bishop, Prakash Satodia, Laura Wild, Jayanta Banerjee, Sian Elliott, Amanda Forster, Albert Demitry, Christina Kortsalioudaki, Amy Woodhead, Heather Barrow, Efygenia Kotsia, Madeleine Barnett, Katharine Thompson

**Affiliations:** a Translational Health Sciences, Bristol Medical School, University of Bristol, United Kingdom; b Population Health Sciences, Bristol Medical School, University of Bristol, United Kingdom; c MRC Integrative Epidemiology Unit, University of Bristol, United Kingdom; d Policy Research Unit in Maternal and Neonatal Health and Care, National Perinatal Epidemiology Unit, Nuffield Department of Population Health, University of Oxford, United Kingdom; e Neonatal Medicine, School of Public Health, Imperial College London, United Kingdom; f Centre for Paediatrics and Child Health, Imperial College, London, United Kingdom; g Department of Population Health Sciences, University of Leicester, United Kingdom

**Keywords:** SARS-CoV-2, COVID-19, Pregnancy, Neonate, Development

## Abstract

**Background:**

Perinatal exposure to SARS-CoV-2 may affect neurodevelopment before 12 months of age, but longer-term outcomes remain unknown. We examined whether antenatal or neonatal SARS-CoV-2 exposure compared with non-exposure is associated with neurodevelopment, respiratory symptoms, and health care usage in early childhood.

**Methods:**

This prospective national population-based cohort study was conducted in England and Wales, United Kingdom. We enrolled term-born children (≥37 weeks' gestation) with and without antenatal or neonatal exposure to SARS-CoV-2 infection by approaching parents of eligible children who were cared for in 87 NHS hospitals. Potential participants were identified through the national active surveillance studies of pregnant women and newborn infants hospitalised with confirmed SARS-CoV-2 infection conducted through the UK Obstetric Surveillance System and the British Paediatric Surveillance Unit. We defined antenatal and neonatal SARS-CoV-2 exposure as infants born to mothers hospitalised with confirmed SARS-CoV-2 infection between 14 + 0 and 36 + 6 weeks gestation and infants admitted to hospital with confirmed SARS-CoV-2 infection within the first 28 days after birth. Children born preterm or with major congenital anomaly or who were not residing in the UK were excluded. We assessed children's development (Ages and Stages Questionnaire 3rd Edition (ASQ-3); Ages and Stages Questionnaire Social-Emotional 2nd Edition (ASQ:SE-2)), respiratory symptoms (Liverpool Respiratory Symptom Questionnaire (LRSQ)) and health care usage (parent-completed questionnaire) at 21–32 months of age. Primary outcome: total ASQ-3 score, converted to z-scores. Secondary outcomes: ASQ:SE-2 z-scores; risk of delay in ASQ-3 domains; total LRSQ scores, converted to z-scores. Analyses were adjusted for children's age, sex, maternal ethnicity, parental education, and index of multiple deprivation.

**Findings:**

Between October 20, 2021 and January 27, 2023, we approached 668 and 1877 families out of 712 and 1917 potentially eligible participants in the exposed and comparison cohort. Of the 125 and 306 participants who were enrolled to the exposed and comparison cohort 121 and 301 participants completed the questionnaires and 96 and 243 participants were included in the analysis. In the age adjusted analysis, the mean total ASQ-3 z-score was lower in the exposed than the comparison cohort (−0.3, 95% CI: −0.6 to −0.05), however, when adjusted for sex, parental education, ethnicity and IMD quintile, there was no significant difference (difference in mean z-score = −0.2 95% CI: −0.5 to 0.03). SARS-CoV-2 exposure was associated with increased risk of delayed personal-social skills (odds ratio = 3.81; 95% CI: 1.07–13.66), higher ASQ:SE-2 total z-scores (difference in mean z-score = 0.4; 95% CI: 0.2–0.6) and increased risk of delayed social-emotional development (OR = 3.58, 95% CI: 1.30–9.83), after adjusting for sex, age at assessment, parental education, ethnicity and IMD quintile. The exposed cohort had a higher mean total LRSQ z-score than the comparison cohort (0.3 95% CI: 0–0.6) and higher inpatient (38% vs. 21%, p = 0.0001), outpatient (38% vs. 30%, p = 0.0090), and General Practitioner appointments (60% vs. 50%, p = 0.021) than the comparison cohort, after adjusting for sex, age at assessment, parental education, ethnicity and IMD quintile. No differences in other secondary outcomes between the exposed and comparison cohorts were found.

**Interpretation:**

Although the exposed cohort did not differ from the comparison cohort on the primary outcome, total ASQ-3 score, the exposed cohort were at greater risk of delayed social-emotional development, had a greater prevalence of respiratory symptoms and increased health care usage relative to the comparison cohort. The study is limited by the smaller sample size due to the low response rate and lack of clinical developmental assessments. Given the association of poor social-emotional development with antenatal or neonatal SARS-CoV-2 exposure, developmental screening, and follow-up of children with confirmed antenatal or neonatal SARS-CoV-2 infection may be warranted to identify those in need of early intervention.

**Funding:**

10.13039/501100000317Action Medical Research for Children.

**Research in context**

**Evidence before this study**

We searched Embase, Emcare, MEDLINE, PsycINFO, Web of Science, and grey literature published to May 8, 2023. Studies were selected if exposure was defined by detection of SARS-CoV-2 during any gestation of pregnancy or in the neonatal period, a contemporaneous non-exposed cohort was examined, and developmental outcomes were reported up to two years of age. No study reported developmental outcomes assessed after 12 months age. A meta-analysis of four studies showed antenatally exposed term-born infants did not have an increased risk of developmental delay. Individually, some studies found lower test scores for fine motor and problem solving and an increased risk of fine motor delay in exposed infants when compared to non-exposed. Developmental outcomes of exposed children assessed beyond 12 months of age and compared with a contemporaneous non-exposed cohort are unknown.

**Added value of this study**

This study, to our knowledge, is the first national, prospective cohort study to show that children exposed to SARS-CoV-2 in the antenatal or neonatal period did not differ on the primary outcome of total ASQ-3 score, however, had an increased odds of delayed social-emotional and personal-social development and higher levels of respiratory symptoms and health care usage across outpatient services, General Practitioner appointments, emergency room visits, and inpatient admissions than the comparison cohort.

**Implications of all the available evidence**

The study is limited by the lower response rate leading to a smaller sample size and lack of clinical developmental assessments. Antenatal or neonatal SARS-CoV-2 exposure may impact children's social-emotional development, level of respiratory symptoms, and health care usage around 2 years of age. These data could inform public health advice and policies for caring for pregnant women and monitoring the development of children exposed to SARS-CoV-2.

## Introduction

Pregnant women with SARS-CoV-2 infection are at increased risk of maternal and neonatal complications.^1^ While neonatal SARS-CoV-2 infection is uncommon, it is associated with adverse neonatal outcomes in some cases.^2^ Even in the absence of severe infection however, in utero exposure to viruses such as Zika has been shown to affect children's brain development and subsequent developmental outcomes. Similarly, SARS-CoV-2 may affect the developing brain through multiple routes including direct invasion, induction of dysregulated autoimmunity with neuroinflammation, or through cerebrovascular factors such as endothelial dysfunction and thrombus formation.^3^ Neurological manifestations of SARS-CoV-2 infection are well described in adults, with concurrent structural, metabolic, and connectivity changes visible on neuroimaging. Although vertical transmission of SARS-CoV-2 is rare,^2^ pregnant women contracting SARS-CoV-2 are prone to developing severe COVID-19 and cytokine storm, and requiring intensive care.^4^ Consequently, there are multiple putative pathways by which brain development during the foetal and neonatal period could be affected by SARS-CoV-2 exposure, with consequent adverse impacts on later neurodevelopment.

To date, several studies have examined development before 12 months of age in infants with prenatal or neonatal SARS-CoV-2 exposure.^5^^,^^6^ These studies used both screening tools and developmental tests but did not consistently identify adverse developmental outcomes associated with prenatal or neonatal SARS-CoV-2 exposure. In contrast, studies that compared the development of children exposed to SARS-CoV-2 during the pandemic with that of children born before the pandemic have reported poorer gross motor, fine motor, and personal social development at 6 months of age,^7^ and poorer communication at 24 months of age.^8^ However, child development is better assessed at or beyond 2 years of age, when measures have greater predictive validity (see research in context). Therefore, there is a need to evaluate longer-term developmental outcomes in children exposed to SARS-CoV-2 antenatally or in the newborn period at or after two years of age compared with non-exposed children also born during the pandemic, to robustly identify any longer-term impact on their development.

SARS-CoV-2 is primarily a respiratory virus. There are some limited data suggesting that antenatal SARS-CoV-2 infection is associated with reduced foetal lung growth.^9^ The respiratory impact of early postnatal infection is more clearly described, for example, infants aged ≤90 days who were SARS-CoV-2 positive have been shown to more frequently presented with cough, upper and lower respiratory symptoms^10^ compared with infants who were SARS-CoV-2 negative. Further, 7.7% (95% CI: 0.9%–25.1%) of children aged <5 years with asymptomatic SARS-CoV-2 infection developed post covid condition presenting predominantly with cough^11^ and there are reports of lung damage in individuals with asymptomatic SARS-CoV-2 infection.^12^ In addition, among children aged 5–18 years who contracted SARS-CoV-2, 27.1% reported long-term respiratory symptoms, however, their pulmonary function did not differ from non-exposed children.^13^ Therefore, it is likely that antenatal or neonatal SARS-CoV-2 exposure could impact the lung development and present with exaggerated respiratory symptoms in early childhood. Given the potential developmental and respiratory sequelae of perinatal SARS-CoV-2 exposure, it is important to understand the influence these sequelae may have on healthcare usage. Therefore, in this study, we aimed to investigate the impact of antenatal and neonatal exposure to SARS-CoV-2 on children's developmental and respiratory outcomes and healthcare usage at two years of age.

## Methods

### Study design and participants

This prospective population-based cohort study recruited children who were born between 1 March 2020 and 28 February 2021 in England and Wales. A detailed protocol for the study was published.^14^ Children were identified through the Urgent Public Health Priority national active surveillance studies of pregnant women and newborn infants hospitalised with confirmed SARS-CoV-2 infection conducted through the UK Obstetric Surveillance System (UKOSS)^15^ and the British Paediatric Surveillance Unit (BPSU)^2^ in the United Kingdom. Participants were recruited from 87 NHS hospitals in England and Wales for this study. Eligible children for the prospective cohort were term-born (≥37 weeks’ gestation) singletons with (exposure cohort) and without (comparison cohort) antenatal or neonatal exposure to SARS-CoV-2 infection. We excluded children who were born preterm, had a major congenital anomaly, or who had left the UK at the time of recruitment. This study adhered to the Strengthening the Reporting of Observational Studies in Epidemiology (STROBE) reporting checklist.

### Procedures

#### SARS-CoV-2 exposure

**Fig. 1:**
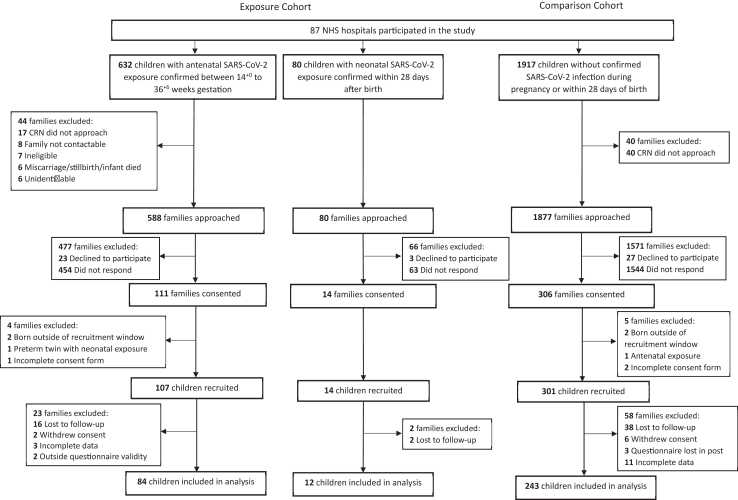
**Cohort flowchart**.

Antenatal exposure was defined as children born to mothers who were hospitalised with confirmed SARS-CoV-2 infection between 14^+0^ and 36^+6^ weeks of gestation. Gestation prior to 14^+0^ was not considered due to the small sample available. Neonatal exposure was defined as infants in hospital with confirmed SARS-CoV-2 infection within the first 28 days after birth, without confirmed SARS-CoV-2 maternal infection in pregnancy. The comparison cohort comprised children without confirmed maternal or neonatal SARS-CoV-2 infection born during the same period as the exposed children (Fig. 1). SARS-CoV-2 exposure was determined by detection of Coronavirus protein (e.g., lateral flow test) or detection of Coronavirus genetic material (e.g., polymerase chain reaction (PCR)). Mothers hospitalised with SARS-CoV-2 infection were classified as having asymptomatic infection, or symptomatic infection with ‘moderate symptoms’ where typical COVID-19 symptoms were present, and ‘severe infection with or without intensive therapy unit (ITU) admission’. Severe maternal infection was categorised as having at least one of the following: <95% oxygen saturation on admission, need for respiratory support, evidence of pneumonia on imaging, or ITU admission.^16^

#### Recruitment

At the sites, NIHR Clinical Research Network teams led by a local principal investigator posted study information and consent forms to the parents of eligible children in the exposure and comparison cohorts. For the comparison cohort, families of the two infants born immediately before and after each child with antenatal SARS-CoV-2 exposure were approached (2–4 comparison children were contacted for each exposed child).

Signed informed consent was sought from parents or carers to approach them when their child reached the assessment age; not all parents who consented for their child to take part in the study completed the assessment.^14^ Data were obtained via questionnaires completed by the parents online (REDCap), by telephone, or by post sent when children reached 21 months of age. This study was approved by the Health Research Authority and London–Westminster Research Ethics Committee (REC ref: 21/PR/0431; protocol no: 2021-93).

#### Developmental assessment

Child development was assessed using the Ages and Stages Questionnaire, 3rd Edition (ASQ-3),^17^ and the Ages and Stages Questionnaire Social-Emotional, 2nd Edition (ASQ:SE-2),^18^ which are validated measures widely used for identifying children at risk of developmental delay.^19^^,^^20^ The ASQ-3 is a parent completed questionnaire that assesses the child's development in five domains: communication, gross motor, fine motor, problem-solving, and personal-social skills. Each domain comprises six questions relating to age-appropriate developmental milestones. Responses are scored as “yes” (score = 10), “sometimes” (5), or “not yet” (0). A total ASQ-3 score is computed ranging from 0 to 300, with higher scores indicating better development. Cut-off scores provided per domain are used to identify children at risk of developmental delay. The ASQ:SE-2 is a 35-item questionnaire used to assess social-emotional development in seven domains: self-regulation, compliance, social communication, adaptive functioning, autonomy, affect, and interaction with people. Responses are scored as “rarely or never” (0), “sometimes” (5), or “most of the time” (10). An additional five points is added per item if the parent/respondent indicates the behaviour is a concern. A total score is calculated from the sum of all the scores, including the additional expressed concern scores, ranging from 0 to 465 with higher scores indicating poorer development. For full details see Supplementary Materials p1.

#### Respiratory symptoms

The Liverpool Respiratory Symptoms Questionnaire (LRSQ) is a validated parent-completed questionnaire^21^ that assesses the frequency of respiratory symptoms within the last three months across eight domains: symptoms when awake, when asleep, during colds, during intervals in between colds, when active; other problems; impact on child; impact on family. Symptom frequency is rated on a five-point Likert scale from “not at all” (0) to “every day” (4). A total LRSQ score is computed ranging from 0 to 32, with higher scores indicating greater respiratory symptoms.

#### Assessment of other health measures

Data pertaining to general health care usage since birth were obtained via a questionnaire developed for this study.

### Outcomes

The primary outcome was the child's total ASQ-3 score. Secondary outcomes were scores below the published age-appropriate cut-off for identifying children at risk of delayed development in each ASQ-3 domain (communication, gross motor, fine motor, problem solving, personal-social, and any domain^17^), total ASQ:SE-2 score, scores above the age-appropriate cut-off on the ASQ:SE-2, and the total LRSQ score.

The following data were collected using a parent-completed questionnaire: child's age, sex, gestational age (GA), birthweight; parental ethnicity; parental education (highest of mother or father for school age qualifications or lower, undergraduate, postgraduate); breastfeeding duration; maternal age at birth; and history of conditions that could impact the child's development (hypoxic-ischaemic encephalopathy, seizures, perinatal stroke, bacterial or non-SARS-CoV-2 viral meningitis, and neuroimaging abnormalities in the neonatal period). We assessed socioeconomic disadvantage using quintile categories from the national Index of Multiple Deprivation (IMD) for England 2019^22^ and for Wales 2019,^23^ derived from the mother's postcode at study entry. Trimester and severity of maternal SARS-CoV-2 infection were obtained from UKOSS data.

### Statistical analysis

We needed a minimum of 85 infants in each of the exposed and comparison cohorts to detect a 0.5 SD difference in mean ASQ-3 total score with a type-I error of 0.05 and 90% power. For all analyses, the antenatal and neonatal exposure group were combined to form the exposed group. Further details are given in the statistical analysis plan.^24^

To assess the representativeness of the participants, baseline characteristics of the antenatal and neonatal exposure cohorts were compared with the eligible cohorts from the UKOSS and BPSU data, and the baseline characteristics of the comparison cohort were compared with national births data for England and Wales from the Office for National Statistics (ONS).^25^ Total ASQ-3 and ASQ:SE-2 scores were converted to z-scores based on the mean and standard deviation in the comparison cohort. A square root transformation was applied to the total LRSQs as these were positively skewed; these transformed scores were converted to z-scores (again, using the mean and standard deviation in the comparison cohort). Multiple linear and logistic regression were used to compare primary and secondary outcomes between exposed and comparison cohorts, adjusting for the following confounders: maternal ethnicity, parental education, and IMD quintile. Due to their strong association with developmental outcomes, infant age and sex were also included in the models to increase precision. Exploratory subgroup analyses were conducted to examine whether an association between SARS-CoV-2 exposure and developmental test scores differs by breastfeeding duration, trimester, or severity of antenatal exposure. A sensitivity analysis evaluated the impact of inclusion of children with other conditions that could impact neurodevelopment. A complete case analysis was performed as the proportion of missing data among responders was low. All analyses were conducted in Stata.

### Role of the funding source

The funder of the study had no role in study design, data collection, data analysis, data interpretation, or writing of the report. Authors EC, RC, RJ had access to the data set and EC had responsibility for the decision to submit for publication.

## Results

### Baseline characteristic comparisons

Between 20th October 2021 and 27th January 2023, we approached 2629 parents from 87 National Health Service Hospitals, of whom 712 were in the exposed cohort (632 antenatal exposure; 80 neonatal exposure) and 1917 in the comparison cohort (Fig. 1). In the antenatal exposure, neonatal exposure, and comparison cohorts, 111/593 (19%), 14/80 (18%), and 306/1877 (16%) parents respectively consented to participate in the study. After exclusions (Fig. 1), 96 children from the exposure cohort (84 antenatal exposure cohort, 12 neonatal exposure cohort) and 243 from the comparison cohort were included in the final analysis. Eligible but not included children (including non-responders, declined consent, lost to follow up) in the exposed cohort were of similar gestational age to the study sample, but differed by maternal ethnicity and sex, with a lower prevalence of White and higher prevalence of Black, mixed, or other ethnicity and male sex in the group not included in the study (Supplementary Materials p2, S1). Eligible but not included children in the comparison cohort were comparable to ONS data for gestational age, but there was a lower prevalence of younger mothers, non-white, and those from more deprived backgrounds (Supplementary Materials p2, S2).

Characteristics of the study population are described in Table 1. The mean (SD) age at assessment was similar between the exposed and comparison cohort. The exposed cohort had a greater proportion of girls than the comparison cohort (58% vs. 45%). Of the exposed mothers 64% were White, 26% were Asian, 10% were Black, mixed, or another race, compared with 91%, 6%, and 3%, respectively, in the comparison cohort. A lower proportion in the exposed cohort had at least one parent educated to degree-level (78% compared with 84% in the comparison cohort). The distribution of socioeconomic disadvantage was similar between cohorts. Breastfeeding beyond 6 months of age was lower in the exposed cohort compared with the comparison cohort (52% vs. 62%). Proportions of missing data in the baseline characteristics were comparable between cohorts (Supplementary Materials p3, S3).

**Table 1:** Baseline characteristics of the SARS-CoV-2 exposed and comparison cohorts among complete cases^a^ (N = 339); figures given are n (%) unless otherwise specified.

Characteristic	Exposed N = 96	Comparison N = 243
Age at assessment (months), mean (SD)	23 (1.7)	22 (1.5)
Child sex		
Male	40 (42%)	134 (55%)
Female	56 (58%)	109 (45%)
Child gestational age, mean (SD)	39 (1.2)	39 (1.2)
Child birthweight (grams), mean (SD)	3228 (539.8)	3430 (451.8)
Maternal age at birth (years), mean (SD)	32 (4.8)	33 (4.7)
Maternal ethnicity		
White	61 (64%)	220 (91%)
Asian	25 (26%)	15 (6%)
Black, mixed, other	10 (10%)	8 (3%)
Paternal ethnicity^b^		
White	63 (67%)	206 (88%)
Asian	21 (22%)	17 (7%)
Black, mixed, other	10 (11%)	11 (5%)
Parental education		
School age qualification or equivalent	22 (23%)	38 (16%)
Undergraduate degree/diploma	37 (39%)	89 (36%)
Postgraduate degree or doctorate	37 (39%)	116 (48%)
Index of multiple deprivation (quintiles)		
1 (least deprived)	16 (17%)	54 (22%)
2	20 (21%)	48 (20%)
3	23 (24%)	51 (21%)
4	18 (19%)	52 (21%)
5 (most deprived)	19 (20%)	40 (16%)
Duration of breastfeeding		
Never/less than one month	31 (32%)	69 (29%)
1–5 months	15 (16%)	23 (10%)
6+ months	50 (52%)	148 (62%)
Conditions that could impact children's neurodevelopment		
Yes	2 (2.1%)	2 (0.8%)
Trimester of antenatal exposure		
2nd	18 (21%)	NA
3rd	66 (79%)	NA
Severity of antenatal exposure		
Asymptomatic	14 (17%)	NA
Symptomatic	45 (54%)	NA
Severe infection/ITU admission	25 (30%)	NA

a Complete cases were defined as those with non-missing ASQ-3 scores, sex, age at assessment, IMD, parental education, and maternal ethnicity [numbers with missing data in exposed cohort: age (n = 1), parental education (n = 5), ASQ-3 (n = 2); in comparison cohort: age (n = 4), parental education (n = 3), IMD (n = 1), ASQ-3 score (n = 5)—see Supplementary Table S3].

b Some individuals had data about maternal but not paternal ethnicity. Since maternal and paternal ethnicity was the same for 92% of the children for whom both were available, we adjusted for maternal ethnicity to maximise the number of complete cases.

### Primary outcomes

On average, ASQ-3 total scores were lower in the exposed cohort than the comparison cohort, although this difference was attenuated after adjusting for confounders (difference in mean z-score (SMD) −0.2; 95% CI: −0.5 to 0.03; p = 0.078) (Table 2).

**Table 2:** Comparison of ASQ-3,^a^ ASQ:SE-2^b^ and LRSQ^c^ between SARS-CoV-2 exposed and comparison cohorts among complete cases (N = 339).

Outcome	Exposed cohort N = 96	Comparison cohort N = 243	Difference in mean z-score/odds ratio (95% CI), adjusted for age	Fully adjusted^d^ difference in mean z-score/odds ratio (95% CI); p-value
**ASQ-3**				
Total score—mean (SD)	240 (42)	249 (32)	−0.3 (−0.6, −0.05)	−0.2 (−0.5, 0.03); p = 0.078
**Below cut-off**—**n (%):**				
Communication	7 (7.3%)	14 (5.7%)	1.16 (0.44, 3.06)	0.99 (0.32, 3.02); p = 0.98
Gross motor	8 (8.3%)	12 (4.9%)	1.91 (0.72, 5.06)	1.78 (0.63, 5.06); p = 0.28
Fine motor	4 (4.2%)	4 (1.6%)	2.46 (0.60, 10.20)	1.04 (0.21, 5.21); p = 0.97
Problem solving	4 (4.2%)	4 (1.6%)	2.51 (0.61, 10.40)	2.72 (0.62, 11.90); p = 0.19
Personal-social	6 (6.3%)	6 (2.5%)	2.81 (0.87, 9.05)	3.81 (1.07, 13.66); p = 0.040
Any domain	16 (16.7%)	30 (12.4%)	1.36 (0.68, 2.68)	1.19 (0.55, 2.58); p = 0.66
**ASQ:SE-2**^e^				
Total score—mean (SD)	32 (35)	19 (23)	0.5 (0.2, 0.7)	0.4 (0.2, 0.6); p = 0.0014
Above cut-off—n (%)	14 (15.2%)	11 (4.5%)	3.65 (1.58, 8.42)	3.58 (1.30, 9.83); p = 0.013
**LRSQ**—median (IQR)				
Day-time symptom score	2 (0, 4)	1 (0, 3)	–	–
Night-time symptom score	3 (1, 5)	2 (1, 4)	–	–
Cold symptom score	2 (1, 6)	2 (1, 4)	–	–
Between colds symptom score	0 (0, 1)	0 (0, 0)	–	–
Activity symptoms score	0 (0, 1)	0 (0, 0)	–	–
Other symptom score	0 (0, 0)	0 (0, 0)	–	–
Effect on child score	0 (0, 3)	0 (0, 2)	–	–
Effect on family score	0 (0, 3)	0 (0, 2)	–	–
Total LRSQ score^f^	10 (3, 24)	8 (3, 18)	0.1 (−0.1, 0.4)	0.3 (0.0, 0.6); p = 0.036

a Ages and Stages Questionnaire, 3rd Edition. ASQ-3 published cut-off for developmental delay.

b Ages and Stages Questionnaire Social-Emotional, 2nd Edition. ASQ:SE-2 published cut-off for developmental delay.

c Liverpool Respiratory Symptom Questionnaire.

d Adjusted for child sex and age in months at assessment, parental education (higher of maternal and paternal; maternal education if paternal education missing), maternal ethnicity, and IMD quintile.

e Seven of the complete cases for the primary analysis (total ASQ-3 score) had missing data for the ASQ:SE-2 and three children had ASQ:SE-2 data but no ASQ-3 data, so the total sample size for this analysis was N = 335, 92 exposed and 243 comparison).

f Square root transformation applied to total score as scores were positively skewed; z-scores were calculated from these transformed scores.

### Secondary outcomes

**Fig. 2:**
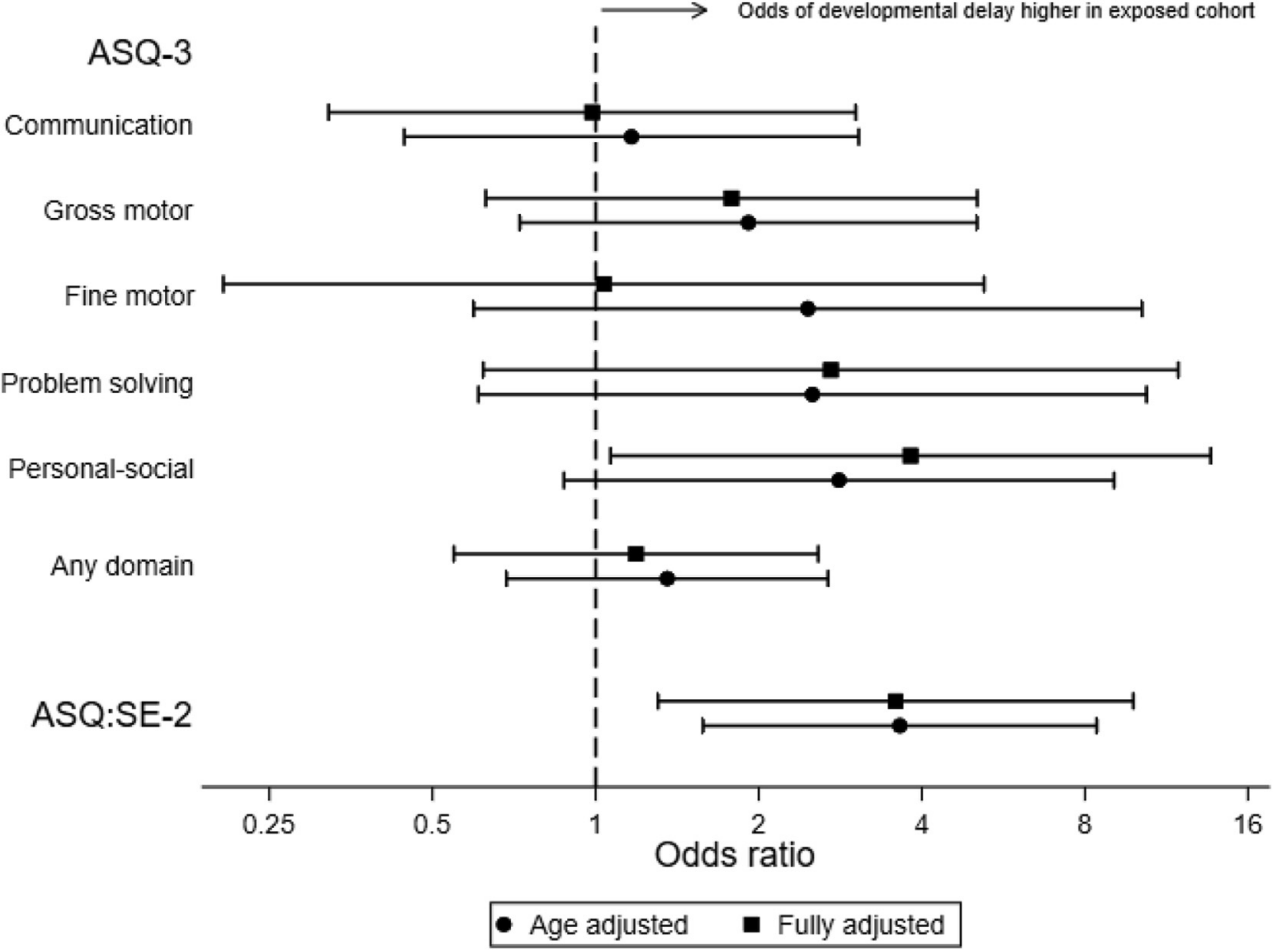
Forest plot of developmental delay in any domain or individual domains of ASQ-3 and ASQ:SE-2 in the exposed compared to comparison cohort. Fully adjusted model included child sex and age in months, parental education (higher of maternal and paternal; maternal education if paternal education missing), maternal ethnicity, and IMD quintile.

In the fully adjusted models, no association was identified between exposure to SARS-CoV-2 infection and risk of delay in communication (OR, 0.99; 95% CI: 0.32–3.02; p = 0.98), gross motor (OR, 1.78; 95% CI: 0.63–5.06; p = 0.28), fine motor (OR, 1.04; 95% CI: 0.21–5.21; p = 0.97), problem-solving (OR, 2.72; 95% CI: 0.62–11.90; p = 0.19), or in any domain (OR, 1.19; 95% CI: 0.55–2.58; p = 0.66) of the ASQ-3. However, SARS-CoV-2 exposure was associated with increased risk of delayed personal-social development (OR: 3.81; 95% CI: 1.07–13.66; p = 0.040) (Table 2 and Fig. 2) and higher ASQ:SE-2 total z-scores (SMD, 0.4; 95% CI: 0.2–0.6; p = 0.0014), indicating greater problems in social-emotional development. Correspondingly, the proportion of children at risk of social-emotional delay was greater in the exposed cohort (OR, 3.58, 95% CI: 1.30–9.83; p = 0.013) (Table 2 and Fig. 2). Total LRSQ z-scores were higher (corresponding to higher number of respiratory symptoms), on average, in the exposed compared with the comparison cohort (mean difference in z-score = 0.3; 95% CI: 0.0–0.6; p = 0.036). The median (IQR) respiratory day-time and night-time symptom score were higher in the exposed than the comparison cohort. Scores for symptoms during colds, between colds, when active, at other times, and for the effect on child and family did not differ substantially between groups (Table 2).

### Subgroup and exploratory analyses

There was no evidence of a difference in ASQ-3 total scores by trimester or severity of infection (Table 3). Duration of breast feeding appear to interact with total ASQ-3 score between exposed and comparison cohort, with having breast fed for more than 6 months was associated with lower total ASQ-3 score in the exposed compared with comparison cohort (p-value for interaction = 0.043; Supplementary Materials p3, S4). Excluding children with other conditions that could impact neurodevelopment (n = 4) gave similar results to those observed in all children (Supplementary Materials p3, S5). ASQ-3 scores were similar when exposure occurred in the antenatal period or neonatal period (Supplementary Materials p3, S6). The exposed and comparison cohorts varied in their use of health care services since birth. On average, the exposed cohort compared with the comparison cohort had a higher proportion of inpatient admissions (38% vs. 21%, p = 0.0001), outpatient (38% vs. 30%, p = 0.0090) and General Practitioner appointments (60% vs. 50%, p = 0.021), accident and emergency visits (57% vs. 51%, p = 0.12), and were visited by a health visitor more regularly outside of routine health checks (9% vs. 3%, p = 0.073). Both cohorts had similar engagement with paediatricians and allied health professionals such as physiotherapists, social workers, opticians, speech and language therapists, dieticians, and others. Personal financial costs and time off work because of the child's health were similar (Supplementary Materials p4, S7).

**Table 3:** ASQ-3^a^ total score by trimester and severity of exposure for the antenatally exposed cohort among complete cases (N = 84).

Outcome	N	Mean (SD) ASQ-3 score	Difference in mean z-score (95% CI), adjusted for age	Fully adjusted^b^ difference in mean z-score (95% CI)
**Trimester**^c^—mean (SD)				
2nd	18	238 (50)	0.04 (−0.8, 0.8)	0.2 (−0.7, 1.0)
3rd	66	241 (43)	Ref	Ref
**Severity**^d^—mean (SD)				
Asymptomatic	14	248 (32)	Ref	Ref
Symptomatic	45	237 (45)	−0.3 (−1.2, 0.6)	−0.2 (−1.2, 0.7)
Severe infection/ITU admission	23	243 (49)	−0.08 (−1.1, 0.9)	0.1 (−0.9, 1.2)

a Ages and Stages Questionnaire, 3rd Edition.

b Adjusted for child sex and age in months at assessment, parental education (higher of maternal and paternal; maternal education if paternal education missing), maternal ethnicity, and IMD quintile.

c p-value = 0.7 in fully adjusted model.

d p-value = 0.6 in fully adjusted model.

## Discussion

In this population based prospective cohort study across England and Wales, there was no significant difference in the primary outcome, total ASQ-3, between the exposed and the comparison cohorts after adjusting for the confounders. We identified that antenatal or neonatal exposure to SARS-CoV-2 infection, when compared with non-exposure, was associated with poorer social-emotional development at two years of age, in terms of both a difference in scores and increased risk of delay. On the ASQ:SE-2, exposed children scored 0.4 SD higher than the comparison cohort (indicating poorer development) and had an increased odds of delayed social-emotional skills compared with the comparison cohort. Similarly, for the ASQ-3, exposed children had an increased odds of delayed personal-social development compared with children in the comparison cohort. Global development, as measured by the total ASQ-3 score, was lower in the exposed cohort than the comparison cohort, although these differences were small and attenuated after adjustment for confounders. This difference was driven by poorer personal-social development as we did not find evidence of differences between the exposed and comparison cohorts in the other domains assessed by the ASQ-3, namely communication, gross motor development, fine motor development, or problem-solving.

Increased risk of delay in both personal social and social-emotional development is a potentially important finding as these both measure the same construct and the results were consistent across the two different scales, ASQ-3 and ASQ:SE-2. A 0.4 SD difference in the social-emotional development between the exposed and comparison cohort is clinically important. For example, a 0.4 SD difference would equate to a 6-point difference in IQ which is clinically relevant on both an individual and population level and has long-term implications. Our findings are further supported by higher rates of positive screening for social-emotional delay identified in a pandemic exposure cohort at 12–18 months of age when compared with a pre-pandemic cohort.^26^ Previous studies have shown that prenatal maternal infections are linked to an increased odds of social-emotional difficulties and emotional symptoms in pre-school children,^27^ and neurodevelopmental conditions^28^ identified throughout childhood such as autism spectrum disorder, attention-deficit/hyperactivity disorder (ADHD) and conduct disorder. Animal studies further support that maternal immune activation is a strong risk factor for abnormal brain development and behavioural difficulties in the offspring.^29^ Although antenatal SARS-CoV-2 infection does not result in changes in maternal immune markers,^30^ neonates exposed to SARS-CoV-2 in the antenatal period had a global cytokine response with upregulation of cytokines including IL-1β, IL-6 and in particular IL-8. Upregulation^31^ of these proinflammatory cytokines are associated with brain damage^32^ and epilepsy.^33^ Our study indicates that antenatal or neonatal exposure to SARS-CoV-2 is associated with an increased risk of social-emotional difficulties in early childhood. Other mediating mechanisms might be important; for example, it is not known how mothers' mental health was impacted by SARS-CoV-2 infection, and specifically how infection affected mother–infant interactions in the exposed cohort. Such indirect impacts may be important because repetitive and synchronic maternal–infant interactions establish the framework for socio-emotional development mediated by oxytocin-induced brain maturation and function.^34^ We did not collect data about mother–infant interaction or postnatal depression in our study. Therefore, the impact on the personal-social and social-emotional development we observed in the exposed cohort could be due to a combination of adverse neonatal inflammation and maternal–infant interactions. Social-emotional delay in infancy poses a risk for persistent social and behavioural problems in childhood and adolescence,^28^ impacting children's ability to develop positive peer relationships and achieve academic success and increasing the risk for ongoing internalising and externalizing behaviour problems.

We observed a small increase in total respiratory symptom score in the exposed compared with the comparison cohort; however, the individual symptoms did not differ substantially between the cohorts, suggesting that lung development may not have been greatly impacted by antenatal or neonatal SARS-CoV-2 infection exposure. This is consistent with data from children aged 5–18 years after SARS-CoV-2 infection. Their pulmonary function was not impaired although 27.1% had persistent respiratory symptoms for 6 months after the infection.^13^ We did, however, find increased overall health care usage among exposed children, which was seen across outpatient and General Practitioner appointments, inpatient admissions, and Emergency room visits. This pattern of predominantly acute health care usage is unlikely to be driven by the developmental impact of SARS-CoV-2 exposure and will need further research to elucidate the reasons for this.

We found no evidence that severity of SARS-CoV-2 exposure was associated with children's development at two years of age, as only small differences were observed between total ASQ-3 scores following asymptomatic, moderate, or severe symptoms of maternal SARS-CoV-2 infection. Although numbers in the severe and asymptomatic antenatal exposure groups were relatively few, meaning that these results should be interpreted cautiously, this contrasts with prior findings that antenatal maternal infections (viral and bacterial) which require hospital admission have stronger associations with poor neurodevelopmental outcomes than milder infections,^35^ and animal models showing maternal immune activation has a dose–response effect on foetal brain development.^28^^,^^29^

Prior research on impact of SARS-CoV-2 exposure on infant neurodevelopment have been inconsistent with no studies beyond two years of age, when developmental assessment is more precise, or lack a contemporary comparison cohort making it challenging to disentangle the effects of SARS-CoV-2 exposure from secondary effects of lockdown policies^36^ and mask wearing^37^ during the COVID-19 pandemic. Our study addresses these gaps by undertaking developmental assessment at 21–32 months of age following SARS-CoV-2 infection compared to a contemporaneous non-exposed cohort. Stratifying the results by trimester of exposure, symptom severity, and breastfeeding duration provides insight into the factors that may influence the impact of maternal/neonatal infection on neurodevelopment. Importantly, our findings do not support previous evidence that exposed infants are at risk of motor and language delay.^38^ Possible reasons include that developmental delay in these domains is reliably identified beyond two years of age, that the effect of exposure on motor development in early infancy may be transient, or that our study included a contemporaneous comparison cohort. Longer-term follow-up is needed to determine whether there are later effects on motor function and/or development in other domains, including social-emotional development.

There are limitations to our study. Our study had a low response, with differences in the characteristics of the studied cohort compared with the eligible cohort, which may impact the generalisability of our findings. Replication of our findings with a larger, more representative cohort is warranted. We restricted the study inclusion criteria to include the antenatal SARS-coV-2 exposure between 14^+0^ and 36^+6^ weeks gestation to investigate exposure before term, however the impact of antenatal SARS-CoV-2 exposure beyond 37 weeks gestation on children's development remain unexplored. Although we adjusted for the key confounding variables, there may still be residual confounding, such as number of siblings, impacting the associations observed between the exposure and outcomes. We did not examine the effect of SARS-CoV-2 exposure beyond the neonatal period in the exposed and comparison cohort, nor did we examine potential neuroprotective influences such as maternal efficacy and parental bonding.^39^ We did not assess the impact of asymptomatic infection in mothers in the comparison cohort on the outcomes. However, such an exposure in the comparison cohort would likely have reduced the observed between-group effect size. Development was assessed using the ASQ-3 suite of instruments. Although these can be used to identify children at risk of developmental delay, screening tools used in early childhood may have poor predictive validity for later outcomes. Therefore, longer term follow-up is needed to determine the persistence of effects identified at two years of age. Another limitation is our small number of neonates with exposure to SARS-CoV-2 such that we were unable to examine whether antenatal or neonatal exposure has differential effects on neurodevelopment. There is a paucity of data concerning developmental outcomes of children exposed to SARS-CoV-2 in the neonatal period and more research is warranted in this population. Finally, while our study was robust to detect a clinically meaningful difference between the exposed and comparison cohorts,^14^ our sensitivity analyses examining trimester and severity of antenatal exposure were limited by the small sample size per subgroup. Future research using a larger cohort of children with antenatal exposure to SARS-CoV-2 would be required to draw more robust conclusions regarding the impact of timing and severity of infection exposure in pregnancy on developmental outcomes.

To conclude, we observed no difference in the primary outcome, total ASQ-3 score, between the exposed and comparison cohorts, after adjusting for confounders, indicating no between-cohort difference in overall development. However, in secondary outcomes, children with antenatal or neonatal exposure to SARS-CoV-2 infection were more likely to score above the cut-off for delayed personal-social development and social-emotional difficulties at two years of age indicating that they might be at increased risk for problems in this area of development. In addition, children with antenatal or neonatal exposure to SARS-CoV-2 infection had higher levels of respiratory symptoms, and greater health care usage at two years of age. If the associations are causal, our findings suggest a need for prenatal/neonatal screening for SARS-CoV-2 infection to identify children at risk and to implement early intervention to mitigate against the potential deleterious effects on social-emotional development for those who need it. This study highlights the need for additional studies to confirm our observed association and for potentially longer-term follow-up of children with exposure to SARS-CoV-2 to monitor their developmental progress and determine whether difficulties in the first two years of life persist later in childhood.

## Contributors

Authors EC, SJ, CG, JK, MK, and RC contributed to the study concept and design. KW, ZD, MH, and RJ were responsible for the acquisition of data. RC did the statistical analyses. EC, SJ, JK, MK, RC, and RJ contributed to the analysis and interpretation of data. EC and RJ wrote the first draft of the manuscript and SJ, CG, JK, RC, MH, KW, ZD critically revised the manuscript and approved the final version. EC, RC, and RJ have access to and verify the underlying study data. EC obtained the funding along with SJ, CG, MK, RC and JK. All authors approved the final text and the decision to submit for publication.

## Data sharing statement

Anonymised participant data that underlie the results reported in this Article can be shared upon specific requests and subject to appropriate approvals. Requests should be directed by email to the corresponding author.

## Declaration of interests

All authors have completed the ICMJE uniform disclosure form. MH, MK received grants from the NIHR in relation to the submitted work; EC, RC, SJ, MK, CG, JK received grant from the Action Medical Research for Children for this work. CG is general secretary and president of The Neonatal Society, and has received grants from the NIHR, Chiesi Pharmaceuticals, Canadian Institute for Health Research (CIHR), and Medical Research Council for neonatal research; no other relationships or activities that could appear to have influenced the submitted work.

## Appendix A Supplementary data

Supplementary Materials

SINEPOST Collaborative group
