# Supplementary material for: Association of antenatal or neonatal SARS-COV-2 exposure with developmental and respiratory outcomes, and healthcare usage in early childhood: a national prospective cohort study
Source: eClinicalMedicine. 2024 May 3;72:102628. doi: 10.1016/j.eclinm.2024.102628 (PMC11087703; doi:10.1016/j.eclinm.2024.102628)
Supplement: Supplementary Materials [file mmc1.docx]

**Supplementary Materials**

**ASQ-3**

Scores corresponding with 2 standard deviations (SD) below the mean point to a need for an immediate referral for further assessment. The ASQ-3 has been shown to have a high negative predictive value (83-89%) at 12 months in screening for motor problems^1^ and a good ability to identify children at risk of developmental problems.^2^ We initially aimed to use the ASQ-3 22-month questionnaire valid for assessment of children aged 21 months 0 days through to 22 months 30 days; however, due to delays in recruitment we also administered the 24-month questionnaire, valid for children aged 23 months 0 days to 25 months 15 days, and the 27-month questionnaire valid for children aged 25 months 16 days to 28 months 15 days.

**ASQ:SE-2**

The tool has generally high internal consistency and test-retest reliability and acceptable sensitivity (range: 0·75-0·89) and specificity (range: 0·82-0·96).^3^ Two versions of the questionnaires were administered: the 24-month (valid at 21 months 0 days to 26 months 30 days) and 30-month (27 months 0 days to 32 months 30 days) versions.

**Supplementary Tables**

**Table S1: Characteristics of the exposed sample compared with all those in the original UKOSS and BPSU studies**

| Characteristic | Analysis sample (n=96) | Non-responders^1^  (n=573) | p-value |
| --- | --- | --- | --- |
| Gestational age  37-38  39-40  41-42  Missing | 47%  48%  5%  0% | 49%  42%  8%  0·2% | P=0.43 |
| Maternal ethnicity  White  Asian  Black/mixed/other  Missing | 64%  26%  10%  0% | 44%  29%  25%  0.2% | P=0.00072 |
| Sex  Male  Female  Missing | 42%  58%  0% | 51%  48%  0·2% | P=0.070 |

**^1^** 668 families were approached. 96 infants were included in the study, leaving 572 infants + 1 infant with incomplete consent form =573 infants in the eligible but not included group. This group was termed as non-responders and include potential participants who did not respond, declined to participate, lost to follow up, withdrew consent, incomplete data or consent form or outside questionnaire validity.

**Table S2: Characteristics of the comparison sample compared with national births data from the Office of National Statistics, England and Wales (ONS) during the study period, 2020-2021.**

| Characteristic | Analysis sample (n=243) | ONS births data |
| --- | --- | --- |
| Gestational age^1^  37-38  39-40  41-42 | 24%  66%  10% | 26%  58%  16% |
| Maternal age  < 25  25-34  35+ | 5%  60%  34% | 14%  61%  25% |
| Maternal ethnicity  White  Asian  Black/mixed/other | 91%  6%  3% | 73%  13%  14% |
| IMD quintile  1 (least deprived)  2  3  4  5 (most deprived) | 22%  19%  21%  21%  16% | 16%  18%  20%  22%  24% |

1. Percentages for ONS data are among those born at term (37 completed weeks gestation or later)

**Table S3: Number (%) with missing data by exposure**

| Variable | Exposed Cohort  (n=101) | Comparison  Cohort  (n=254) |
| --- | --- | --- |
| Child age at assessment (months) | 1 (1·0%) | 4 (1·6%) |
| Child sex | 0 | 0 |
| Maternal ethnicity | 0 | 0 |
| Paternal ethnicity | 4 (4·0%) | 9 (3·5%) |
| Parental education (higher of maternal/paternal) | 5 (5·0%) | 3 (1·2%) |
| Index of multiple deprivation | 0 | 1 (0·4%) |
| ASQ-3 | 2 (2·0%) | 5 (2·0%) |
| ASQ:SE-2 | 5 (5·0%) | 5 (2·0%) |

**Table S4: Comparison of total ASQ-3 score between SARS-CoV-2 exposed and comparison cohorts by breastfeeding category**

| Breastfeeding duration | N | Exposed Cohort | N | Comparison Cohort | Difference in mean z-score (95% CI), adjusted for age | Fully adjusted^1^ difference in mean z-score |
| --- | --- | --- | --- | --- | --- | --- |
| Never/<1 month  1-5 months  6+ months | 31 15 50 | 246 (40)  231 (39)  239 (44) | 69 23 148 | 239 (35)  248 (34)  255 (29) | 0·2 (-0·2, 0·7)  -0·7 (-1·5, 0·2)  -0·5 (-0·8, -0·2) | 0·1 (-0·4, 0·7)  -1·2 (-2·1, -0·4)  -0·4 (-0·8, -0·04) |

- 1. Total ASQ-3 score between SARS-CoV-2 exposed and comparison cohorts by breastfeeding category. P-value for interaction = 0.043 in fully adjusted model.

**Table S5: Comparison of total ASQ-3 score and proportion below cut-off between SARS-CoV-2 exposed and comparison cohorts excluding children with other conditions that could impact neurodevelopment.**

| Outcome | Exposed Cohort  N=94^1^ | Comparison Cohort  N=241^1^ | Adjusted difference in mean z-score /odds ratio (95% CI) |
| --- | --- | --- | --- |
| Total score – mean (SD)  Proportion below cut-off in any domain | 241 (41)  16% | 249 (32)  12% | -0·2 (-0·5, 0·08)  1·18 (0·53, 2·61) |

- 1. There were two individuals in each group with conditions that could impact neurodevelopment and a further two in the exposed cohort where this information was missing.

**Table S6: Comparison of total ASQ-3 score by timing of exposure**

| Outcome | Antenatal exposure  N=84 | Neonatal exposure  N=12 | Comparison Cohort  N=243 | Adjusted difference in mean z-score (95% CI) | |
| --- | --- | --- | --- | --- | --- |
|  |  |  |  | Antenatal | Neonatal |
| Total score – mean (SD) | 241 (44) | 237 (23) | 249 (32) | -0·2 (-0·5, 0·06) | -0·3 (-1·0, 0·3) |

**Table S7. Health care usage among the SARS-CoV-2 exposed and comparison cohorts.**

| Variable |  | Exposed Cohort | Comparison Cohort | p-value^1^ |
| --- | --- | --- | --- | --- |
| Hospital admission since birth   - Inpatient - Outpatient | Never  Once  2+ times  Never  Once  2+ times | 55 (62%)  18 (20%)  16 (18%)  56 (62%)  14 (16%)  20 (22%) | 188 (79%)  31 (13%)  20 (8%)  169 (71%)  35 (15%)  35 (15%) | P=0.0001  P=0.0090 |
| Accident and Emergency visit since birth | Never  Once  2+ times | 39 (43%)  20 (22%)  31 (34%) | 116 (49%)  67 (28%)  56 (23%) | P=0.12 |
| Community professional visit outside of routine heath checks   - General Practitioner   Ever visited:   - Health visitor - Practice nurse - Community paediatrician, physiotherapist, social worker, optician, speech and language therapist, dietitian, other | Never  Once  Twice  3+ times  Yes  Yes  Yes | 36 (40%)  19 (21%)  19 (21%)  16 (18%)  8 (9%)  5 (6%)  15 (17%) | 119 (50%)  53 (22%)  35 (15%)  32 (13%)  8 (3%)  9 (4%)  41 (17%) | P=0.021  P=0.073  P=0.36  P=0.96 |
| Personal financial costs related to child’s health   - Any costs? - Median (IQR) costs among those with costs | Yes | 7 (7%)  £150  (£100, £2,500) | 13 (5%)  £225  (£125, £390) | P=0.38 |
| Parents time off work due to child’s health   - Any days off with pay - Median (IQR) number days off among those with any - Any days off without pay - Median (IQR) number days off among those with any | Yes  Yes | 25 (26%)  4 (2, 8)  37 (15%)  5 (2, 11·5) | 95 (39%)  5 (2, 7)  12 (13%)  4 (2, 5) | P=0.43  P=0.82 |

- - 1. P-value obtained from logistic regression or ordinal logistic regression, adjusted for infant’s age, parental education, maternal ethnicity, and IMD quintile.

**Non-author contributors**

| UKOSS group  **Melanie O’Connor,** Programme Manager, National Perinatal Epidemiology Unit, Nuffield Department of Population Health, University of Oxford, UK  **Kudzai Chinjekure,** Data Coordinator, National Perinatal Epidemiology Unit, Nuffield Department of Population Health, University of Oxford, UK  **Nicola Vousden**, Clinical Research Fellow in Maternal and Child Population Health, National Perinatal Epidemiology Unit, Nuffield Department of Population Health, University of Oxford, UK  **Rema Ramakrishnan***,* Senior Statistician, National Perinatal Epidemiology Unit, Nuffield Department of Population Health, University of Oxford, UK    **Kathryn Bunch**, Epidemiologist, National Perinatal Epidemiology Unit, Nuffield Department of Population Health, University of Oxford, UK  **Eddie Morris**, Consultant Gynaecologist, Royal College of Obstetricians and Gynaecologists, London, UK  **Nigel A B Simpson,** Consultant Obstetrician and Gynaecologist, Department of Women’s & Children’s Health, University of Leeds, Leeds, UK  **Patrick O’Brien,** Consultant Obstetrician and Gynaecologist, Royal College of Obstetricians and Gynaecologists, London, UK, Institute for Women's Health, University College London, London, UK  **Peter Brocklehurst,** Professor of Women’s Health, Birmingham Clinical Trials Unit, University of Birmingham, Birmingham, West Midlands, UK |
| --- |
| BPSU group  **Kathryn E Fitzpatrick,** Senior Researcher in Statistical Epidemiology, NIHR Policy Research Unit in Maternal and Neonatal Health and Care, National Perinatal Epidemiology Unit, Nuffield Department of Population Health, University of Oxford, UK  **Helen Mactier**, Neonatal Consultant and Honorary Clinical Associate Professor, Princess Royal Maternity and the University of Glasgow, Glasgow, UK  **Alessandra Morelli**, Research Midwife, NIHR Policy Research Unit in Maternal and Neonatal Health and Care, National Perinatal Epidemiology Unit, Nuffield Department of Population Health, University of Oxford, UK  **Mariko Nakahara,** Research Assistant, NIHR Policy Research Unit in Maternal and Neonatal Health and Care, National Perinatal Epidemiology Unit, Nuffield Department of Population Health, University of Oxford, UK  **Anna Placzek**, Project Manager, NIHR Policy Research Unit in Maternal and Neonatal Health and Care, National Perinatal Epidemiology Unit, Nuffield Department of Population Health, University of Oxford, UK    **Shamez N Ladhani**, Consultant Epidemiologist, Public Health England, Colindale, UK; Professor of Paediatric Infectious Diseases and Vaccinology, St. George’s University of London, UK  **Elizabeth S Draper**, Professor of Perinatal & Paediatric Epidemiology, Department of Health Sciences, University of Leicester, Centre for Medicine, University Road, Leicester, UK  **Don Sharkey**, Professor of Neonatal Medicine and Technologies, Centre for Perinatal Research, School of Medicine, University of Nottingham, UK  **Cora Doherty**, Consultant Neonatologist, University Hospital of Wales, Cardiff, UK  **Maria A Quigley**, Professor of Statistical Epidemiology, NIHR Policy Research Unit in Maternal and Neonatal Health and Care, National Perinatal Epidemiology Unit, Nuffield Department of Population Health, University of Oxford, UK |

**Details of the SINEPOST collaborative group**

Co-investigators and writing committee:

Ela Chakkarapani**,** Associate Professor of Neonatal Neuroscience, Consultant Neonatologist, Translational Health Sciences, Bristol Medical School, University of Bristol, United Kingdom.

Rebecca Jackson, Research Assistant, Translational Health Sciences, Bristol Medical School, University of Bristol, United Kingdom.

Rosie Cornish, Population Health Sciences, Bristol Medical School, University of Bristol, United Kingdom, MRC Integrative Epidemiology Unit, University of Bristol, United Kingdom.

Zoe Daskalopoulou, Policy Research Unit in Maternal and Neonatal Health and Care, National Perinatal Epidemiology Unit, Nuffield Department of Population Health, University of Oxford, United Kingdom.

Chris Gale, Professor of Neonatal Medicine, School of Public Health, Faculty of Medicine, Imperial College London, United Kingdom.

Madeleine Hurd, Policy Research Unit in Maternal and Neonatal Health and Care, National Perinatal Epidemiology Unit, Nuffield Department of Population Health, University of Oxford, United Kingdom.

Samantha Johnson, Professor of Child Development, Department of Population Health Sciences, University of Leicester, United Kingdom.

Marian Knight, Professor of Maternal and Child Population Health, NIHR Policy Research Unit in Maternal and Neonatal Health and Care, National Perinatal Epidemiology Unit, Nuffield Department of Population Health, University of Oxford, UK.

Jennifer J Kurinczuk, Professor of Perinatal Epidemiology, NIHR Policy Research Unit in Maternal and Neonatal Health and Care, National Perinatal Epidemiology Unit, Nuffield Department of Population Health, University of Oxford, UK.

Kathryn Woodward, Research Associate, Translational Health Sciences, Bristol Medical School, University of Bristol, United Kingdom.

Funding committee

Helen Mactier, Neonatal Consultant and Honorary Clinical Associate Professor, Princess Royal Maternity and the University of Glasgow, Glasgow, UK

Elizabeth S Draper, Professor of Perinatal & Paediatric Epidemiology, Department of Health Sciences, University of Leicester, Centre for Medicine, University Road, Leicester, UK

Don Sharkey, Professor of Neonatal Medicine and Technologies, Centre for Perinatal Research, School of Medicine, University of Nottingham, UK

Cora Doherty, Consultant Neonatologist, University Hospital of Wales, Cardiff, UK

Clinical Research Network

George Eliot Hospital NHS Trust: Karen Shorthose
Royal Devon and Exeter NHS Foundation Trust: Nagendra Venkata, Claire Cooper
Norfolk and Norwich University Hospitals NHS Foundation Trust: Claire Lee, Louise Coke
Royal Free London NHS Foundation Trust: Clare Cane, Cynthia Diaba
West Hertfordshire Teaching Hospitals NHS Trust: Dr Sankara Narayanan
Medway NHS Foundation Trust: Ghada Ramadan
North West Anglia NHS Foundation Trust: Alys Capell
Royal United Hospitals Bath NHS Foundation Trust: Dr Dan Jolley, Jennifer Pullen
Bradford Teaching Hospitals NHS Foundation Trust: Rachel Wane, Liz Ingram
St Helens and Knowsley Teaching Hospitals NHS Trust: Rosaline Garr, Amy Millington
North Bristol NHS Trust: Manal El-Bokle, Paula Brock
East Lancashire Hospitals NHS Trust: Bev Hammond, Matthew Milner
University Hospitals of Derby and Burton NHS Foundation Trust: Dr Shalini Ojha, Sarah Miller
University Hospitals Dorset NHS Foundation Trust: Stephanie Grigsby, Susara Blunden
Epsom and St Helier University Hospitals NHS Trust: Dr Ruth Shephard, Dr Emma Williams
University Hospital Southampton NHS Foundation Trust: Balamurugan Thyagarajan, Phillippa Crowley
St George's University Hospitals NHS Foundation Trust: Kirsty Le Doare, Emily Marler
Barts Health NHS Trust: Ajay Sinha, Nicolene Plaatjas
York and Scarborough Teaching Hospitals NHS Foundation Trust: Dr Dominic Smith, Jennifer Baker University Hospitals of Leicester NHS Trust: Muhammad Ali, Jennifer Smith
Manchester University NHS Foundation Trust: Ranganath Ranganne, Kate Stanbury
Portsmouth Hospitals University NHS Trust: Dr Tim Scorrer, Alison LePoidevin
Royal Berkshire NHS Foundation Trust: Sharon Westcar
Cambridge University Hospitals NHS Foundation Trust: Myrna Maquinana
South Warwickshire NHS Foundation Trust: Clare O'Brien, Seren Willson
Nottingham University Hospitals NHS Trust: Don Sharkey, Jessica Simkin
Bedfordshire Hospitals NHS Foundation Trust: Dr Amy Carmichael
Lewisham and Greenwich NHS Trust: Dr Laura Salter, Dr Bhavna Sharma
Mid Cheshire Hospitals NHS Foundation Trust: Caroline Dixon, Janet Brown, Amaryl Jones
Worcestershire Acute Hospitals NHS Trust: Kate Townsend, Catherine Townsend
Bolton NHS Foundation Trust: Emma Tanton
Homerton University Hospital NHS Foundation Trust: Paul Fleming, Fiona Stacey
Liverpool Women's NHS Foundation Trust: Richard Hutchinson, Laura Plummer
Maidstone and Tunbridge Wells NHS Trust: Louise Swaminathan
East and North Hertfordshire NHS Trust: Dr Charu Bhatia, Rebecca Dubber
The Newcastle upon Tyne Hospitals NHS Foundation Trust: Dr Jenny Dixon, Angela Phillipson, Julie Groombridge
Great Western Hospitals NHS Foundation Trust: Tracey Benn
The Leeds Teaching Hospitals NHS Trust: Dr Kathryn Johnson, Lindsay Uryn
Buckinghamshire Healthcare NHS Trust: Dr Sanjay Salgia, Lisa Frankland
Royal Surrey NHS Foundation Trust: Caroline Salmon, Asharee Green
The Hillingdon Hospitals NHS Foundation Trust: Elizabeth Lek, Nerea Rodal-Prieto
Wirral University Teaching Hospital NHS Foundation Trust: Julie Grindey
Northern Care Alliance NHS Group: Grainne O'Connor
Croydon Health Services NHS Trust: Dr Afaf Tebbal, Sophie Cullinan
Lancashire Teaching Hospitals NHS Foundation Trust: Paula Sugden
Doncaster and Bassetlaw Teaching Hospitals NHS Foundation: Dr Umberto Piaggio, Sarah Farmer
Stockport NHS Foundation Trust: Daisy Tudor
Barking, Havering and Redbridge University Hospitals NHS Trust: Dr Ambalika Das, Dr Donna Nicholls
Royal Cornwall Hospitals NHS Trust: Dr Charlotte Lea, Ruth Bowen
Somerset NHS Foundation Trust: Rebecca Mann
Calderdale and Huddersfield NHS Foundation Trust: Georgina Turner
Guy's and St Thomas' NHS Foundation Trust: Chinthika Piyasena, Joanna Robinson
North Middlesex University Hospital NHS Trust: Dr Jain Neeraj, Gillian Godwin, Bridget Oduro
King's College Hospital NHS Foundation Trust: Gillian Godwin
University Hospitals Sussex NHS Foundation Trust: Ramon Fernandez
Royal Wolverhampton NHS Trust: Dr Kalyana Gurusamy
Sheffield Teaching Hospitals NHS Foundation Trust: Liz Pilling
University Hospitals Birmingham NHS Foundation Trust: Richard Mupanemunda
Mid Yorkshire Hospitals NHS Trust: Dr Sarah Didier
Whittington Health NHS Trust: Jessica Ellis
Aneurin Bevan University Health Board: Dr Anitha James
Princess Elizabeth Hospital, Le Vanquiedor: Sandie Bohin
Southport & Ormskirk Hospitals NHS Trust: Linda Bishop
University Hospitals Coventry and Warwickshire NHS Trust: Dr Prakash Satodia, Laura Wild
Imperial college Healthcare NHS Trust: Jayanta Banerjee
Cardiff and Vale University Health Board: Sian Elliott
South Tees Hospitals NHS Foundation Trust: Amanda Forster
Sandwell and West Birmingham NHS Trust: Albert Demitry
University College London Hospitals NHS Foundation Trust: Dr Christina Kortsalioudaki
Birmingham Women's and Children's NHS Foundation Trust: Amy Woodhead, Heather Barrow, Efygenia Kotsia
Chelsea and Westminster Hospital NHS Foundation Trust: Madeleine Barnett
University hospitals Bristol and Weston NHS Foundation Trust: Katharine Thompson

**References**

1 Hwarng, G. Y. H. et al. Accuracy of Parent-Reported Ages and Stages Questionnaire in Assessing the Motor and Language Skills of Preterm Infants. *J Neonatal Perinatal Med* **14**, 193-202 (2021).

2 Steenis, L. J., Verhoeven, M., Hessen, D. J. & van Baar, A. L. Parental and Professional Assessment of Early Child Development: The Asq-3 and the Bayley-Iii-Nl. *Early Hum Dev* **91**, 217-225 (2015).

3 Squires, J., Bricker, D., Heo, K. & Twombly, E. Identification of Social-Emotional Problems in Young Children Using a Parent-Completed Screening Measure. *Early Childhood Research Quarterly* **16**, 405-419 (2001).
