# Supplementary material for: Association of antenatal or neonatal SARS-COV-2 exposure with developmental and respiratory outcomes, and healthcare usage in early childhood: a national prospective cohort study
Source: eClinicalMedicine. 2024 May 3;72:102628. doi: 10.1016/j.eclinm.2024.102628 (PMC11087703; doi:10.1016/j.eclinm.2024.102628)
Supplement: SINEPOST Collaborative group [file mmc2.docx]

**Details of the SINEPOST collaborative group**

Collaborators:

| **First name** | **Last name** |
| --- | --- |
| Helen | Mactier |
| Elizabeth | Draper |
| Don | Sharkey |
| Cora | Doherty |
| Karen | Shorthose |
| Nagendra | Venkata |
| Claire | Cooper |
| Claire | Lee |
| Louise | Coke |
| Clare | Cane |
| Cynthia | Diaba |
| Sankara | Narayanan |
| Ghada | Ramadan |
| Alys | Capell |
| Dan | Jolley |
| Jennifer | Pullen |
| Rachel | Wane |
| Liz | Ingram |
| Rosaline | Garr |
| Amy | Millington |
| Manal | El-Bokle |
| Paula | Brock |
| Bev | Hammond |
| Matthew | Milner |
| Shalini | Ojha |
| Sarah | Miller |
| Stephanie | Grigsby |
| Susara | Blunden |
| Ruth | Shephard |
| Emma | Williams |
| Balamurugan | Thyagarajan |
| Phillippa | Crowley |
| Kirsty | Le Doare |
| Emily | Marler |
| Ajay | Sinha |
| Nicolene | Plaatjas |
| Dominic | Smith |
| Jennifer | Baker |
| Muhammad | Ali |
| Jennifer | Smith |
| Ranganath | Ranganne |
| Kate | Stanbury |
| Tim | Scorrer |
| Alison | LePoidevin |
| Sharon | Westcar |
| Myrna | Maquinana |
| Clare | O'Brien |
| Seren | Willson |
| Jessica | Simkin |
| Amy | Carmichael |
| Laura | Salter |
| Bhavna | Sharma |
| Caroline | Dixon |
| Janet | Brown |
| Amaryl | Jones |
| Kate | Townsend |
| Emma | Tanton |
| Paul | Fleming |
| Fiona | Stacey |
| Richard | Hutchinson |
| Laura | Plummer |
| Louise | Swaminathan |
| Charu | Bhatia |
| Rebecca | Dubber |
| Jenny | Dixon |
| Angela | Phillipson |
| Julie | Groombridge |
| Tracey | Benn |
| Kathryn | Johnson |
| Lindsay | Uryn |
| Sanjay | Salgia |
| Lisa | Frankland |
| Caroline | Salmon |
| Asharee | Green |
| Elizabeth | Lek |
| Nerea | Rodal-Prieto |
| Julie | Grindey |
| Grainne | O'Connor |
| Afaf | Tebbal |
| Sophie | Cullinan |
| Paula | Sugden |
| Umberto | Piaggio |
| Sarah | Farmer |
| Daisy | Tudor |
| Ambalika | Das |
| Donna | Nicholls |
| Charlotte | Lea |
| Ruth | Bowen |
| Rebecca | Mann |
| Georgina | Turner |
| Chinthika | Piyasena |
| Joanna | Robinson |
| Jain | Neeraj |
| Gillian | Godwin |
| Bridget | Oduro |
| Ramon | Fernandez |
| Kalyana | Gurusamy |
| Liz | Pilling |
| Richard | Mupanemunda |
| Sarah | Didier |
| Jessica | Ellis |
| Anitha | James |
| Sandie | Bohin |
| Linda | Bishop |
| Prakash | Satodia |
| Laura | Wild |
| Jayanta | Banerjee |
| Sian | Elliott |
| Amanda | Forster |
| Albert | Demitry |
| Christina | Kortsalioudaki |
| Amy | Woodhead |
| Heather | Barrow |
| Efygenia | Kotsia |
| Madeleine | Barnett |
| Katharine | Thompson |
